# OpenHW3 – An open-source, low-cost temperature-controlled orbital shaker

**DOI:** 10.1016/j.ohx.2024.e00570

**Published:** 2024-08-13

**Authors:** Matthew Hollingham, Yi Xiang, Titus Reed, Juan Pablo Gevaudan

**Affiliations:** Re-AIM Laboratory, The Pennsylvania State University, USA

**Keywords:** Open source, Orbital shaker, Temperature control, Binding isotherms

## Abstract

The current lack of standardized testing methods to assess the binding isotherms of ions in cement and concrete research leads to uncontrolled variability in these results. In this study, an open-source and low-cost apparatus, named OpenHW3, is proposed to accurately measure the binding isotherms of ions in various cementitious material systems. OpenHW3 provides two main options, a temperature-controlled orbital shaker, as well as an option to retrofit a commercial orbital shaker for temperature control. The effectiveness of these device options is validated via comparison with conventional binding isotherms experiments. The binding isotherm results were comparable to conventional Waterbath shakers, while providing more reliable results compared to horizontal commercial shakers. It also provided accurate temperature control between 25 °C and 75 °C. The results here are critical for allowing open access to scientific equipment, and providing high-quality binding isotherm data for reliable service life models of urban infrastructure assets throughout the world.

## Graphical abstract

**Figure:**
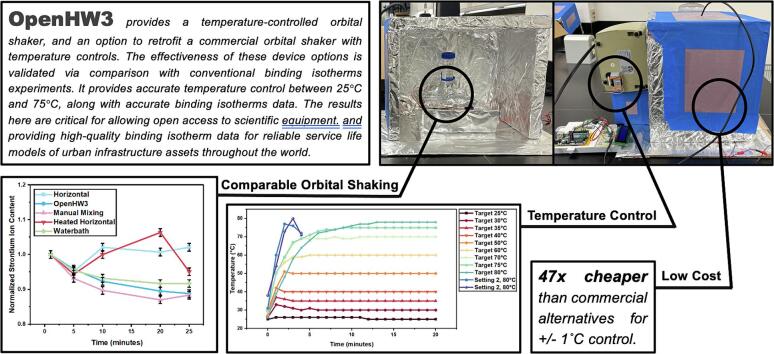

**Specifications table**

**Table:** 

Hardware name	*OpenHW3*
Subject area	• Engineering and materials science • Chemistry and biochemistry • Biological sciences (e.g., microbiology and biochemistry) • General
Hardware type	• Measuring physical properties and in-lab sensors • Biological sample handling and preparation • Mechanical engineering and materials science
Closest commercial analog	*Orbital shaker, temperature controlled orbital shaker, binding isotherms experimental setup.*
Open source license	*License: CC0 1.0 Universal*
Cost of hardware	*$169*
Source file repository	*DOI 10.17605/OSF.IO/8N65F,**https://osf.io/8n65f/*
OSHWA certification UID *(OPTIONAL)*	*US002526*

## 1 Hardware in context

Cement and concrete research [1], [2], [3], [4], along with biomedical [5], [6], [7] and chemical research [8], [9], [10], employ binding isotherm experiments to describe the physi- and chemisorption of ions into material substrates. This experiment is commonly employed in cement and concrete research applications to determine the chloride binding of cement-based materials subjected to external chloride environments, such as marine environments. The most common method for determining chloride binding in this field is the ‘equilibrium method’ [2]. This test relies upon laboratory conditions remaining stable over long periods (14 days to 1 year) to attain a chemical equilibrium between the investigated ions in a solution and the ionic sorption of the material. The timescale variability is due to the particle sizes of the cement. With cement pastes at sizes 10 mm thick requiring up to 1 year [11] for an equilibrium to be reached, while with a reduction of particle size to 0.25–2 mm [12], absorption equilibrium can be reached within 14 days. However, equilibrium may still not be reached, and free chloride ions have been shown to increase after 84 days of immersion at small particle sizes [13].

When completing the binding isotherms experiment with reduced particle sizes (0.25–2 mm), it is common for no mixing to occur, with samples left in a solution [14], [12], [2], [11] until equilibrium is reached. To reduce the time taken to complete the experiment, continuous mixing is employed. This also increases the rate at which equilibrium will be attained [2]. Currently, it is more common to use either intermittent mixing with an orbital shaker at standard laboratory temperature conditions or a temperature-controlled orbital shaker (or water bath shaker) [1], [16], [15], [17], [18]. These two options present a significant rise in the cost of performing binding isotherms experiments due to the cost of scientific temperature-controlled orbital shaking equipment. This cost may present an implementation barrier for laboratories as these systems can cost up to $10,000. These costs (as of November 2023) are summarised in Table 1 for temperature-controlled orbital shakers and Table 2 for non-temperature-controlled orbital shakers, noting around a 5× price increase between non-temperature control and temperature control.

**Table 1:** Summary of commercial orbital shakers with temperature control.

**Manufacturer**	**Product name**	**Orbit**	**Temperature range**	**Tray size**	**Price**
**ThermoFisher**	MaxQ 4450 Mini Benchtop Shaker	1.9 cm	Ambient +5 to 60 °C	28 × 33 cm	$7,829.00 + $119.00 shipping
**ThermoFisher**	MaxQ 420 Incubated Tabletop High Performance Shaker	2.5 cm	Ambient +5 to 80 °C	53.8 × 54.4 × 32 cm	$11,496.00 + $119 shipping
**Avantor**	VWR® Incubating Orbital Shaker, Model 5000I	2.54 cm	Ambient +5 to 65 °C	45 × 45 cm	$12,171.28
**OHAUS**	ISHD23HDG	25 mm	Ambient +5 to 65 °C	457 × 457 mm	$7,506 + $80 Shipping
**Benchmark Scientific**	Incu-Shaker™ Mini	19 mm	Ambient +5 to 70 °C	28 × 41 × 33 cm	$3,298
**Scientific Industries**	Genie Temp-Shaker 300	19 mm	28 to 75 °C	305 × 305 mm	$2,677
**New Brunswick™**	Excella® E24 Series	19 mm	Ambient +7 to 60 °C	46 × 46 cm	$7,710
**Scientific Industries Inc**	Incubator-Genie	Rotate and shake	28 to 75 °C	8′’ × 12′’	$4,320
**Jeio Tech**	IST Series Incubated Shakers	19.1 mm	Ambient +5 to 80 °C	350 × 350 mm	$6,278
**Sheldon Manufacturing**	SSI3 Benchtop Shaking Incubator	Unspecified	Ambient +8 to 60 °C	44 × 45 cm	$8,161

**Table 2:** Summary of commercial orbital shakers.

	**Product**	**Orbit**	**Tray size**	**Max capacity**	**Price**
**Benchmark Scientific**	Orbi-Shaker™ JR.	19 mm	28 × 27 × 13 cm	4 kg	$1,084
**Scilogex**	SCI-O180-S Digital Orbital Shaker	20 mm	12 × 9 inches	3 kg	$589
**Benchmark Scientific**	Orbi-Blotter™	19 mm	35 × 30 cm	4 kg	$921
**IKA**	KS 260 Basic/Control	10 mm	36 × 42 cm	7.5 kg	$3,352
**Jeio Tech**	OS-2000	12.7 mm	320 × 260 mm	8.5 kg	$2,121
**Ohaus**	Digital Light Duty Orbital Shakers	15 mm	11.75 × 8.75 in.	3.6 kg	$1,577
**BEING**	BS Series Orbital Shakers	20 mm	10 × 10 inches	2 kg	$1,100
**New Brunswick™**	Innova® Open air Series	1.9 cm	33 × 28 cm	Not specified	$3,187
**Scientific Industries**	Mini-100 Orbital-Genie	19 mm	0.9 sq ft	6.6 lbs	$929
**Jeio Tech**	Mini Shaker CMS-350	13 mm	276 × 276 mm	5.2 kg	$1,627
**IKA**	KS 130 Basic	4 mm	320 × 260 mm	2 kg	$2,248
**Open-source**	DIYBio	19 mm	5 × 5 cm	Not specified	$138.22 plus shipping and 5–7 h build time (Total estimate $250)

The high costs associated with non-temperature and temperature-controlled orbital shakers has motivated the development of open-source alternatives for orbital shakers [19], [20]. Recently, Baillargeon et al. developed an an open-source orbital shaker working in a 2-year period in laboratory temperature and humidity conditions [21]. With this, open-source temperature control of equipment has also been attempted for other laboratory purposes [22], and a large market exists for large-scale temperature-controlled chambers [21]. However, these open-source alternatives have design issues, such as 3D printed parts failure over long use periods and loosening of parts [21]. These develop mainly due to the weak layer-by-layer nature of 3D printed parts. Additionally, long-term damage from placing microcontrollers inside environmental chambers with high humidity and temperature conditions has been observed. These issues are not commonly observed for commercial orbital shakers.

Another large factor when choosing between and open-source option and a commercial alternative is time. With an open-source option, there is trade off with a product that once bought, will more-reliably work once it arrives to a laboratory. From the current open-source options explained above, these all require multiple parts from various vendors, a 3D printer, and 5–7 h of assembly time. Although having a lower cost than their commercial alternatives, most of these published options do not consider limitations associated with part delivery costs, part shipping time, 3D printing errors, the time taken to order the parts, equipment needed for assembly (drills and soldering irons), materials required to assemble it (glues and 3D filaments) and other stages of the assembly that may result in manufacturing errors. When considering these limitations in the context of time and labour expenses, the benefits of open-source hardware may be significantly diminished. Thus, there is a critical need to develop efficient, low-part number, simple designs for heated orbital shaker alternatives.

## 2 Hardware description

**Fig. 1:**
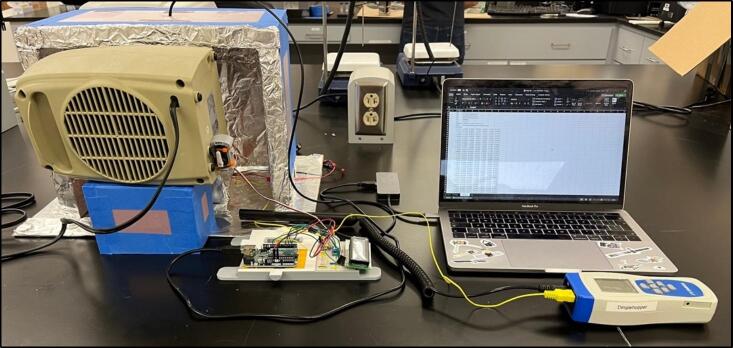
OpenHW3 with 3D printed orbital shaker provided.

**Fig. 2:**
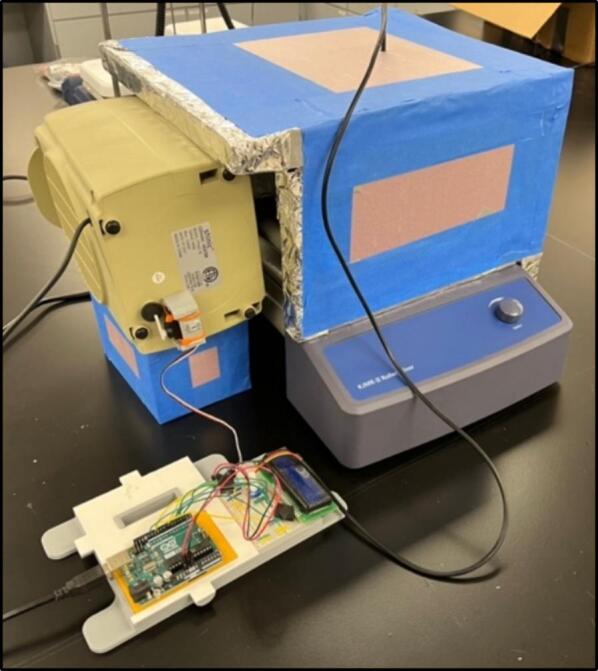
OpenHW3 with commercial orbital shaker.

To solve these limitations, OpenHW3 addresses two main challenges, namely, a complete temperature-controlled orbital shaker, and a method to heat an existing laboratory orbital shaker from ambient to 75 °C. It consists of an insulant and aluminium foil structure, an optional 3D-printed orbital shaker, and an Arduino-controlled heater fitted with an LCD display. These designs are displayed in Fig. 1, Fig. 2.

**Fig. 3:**
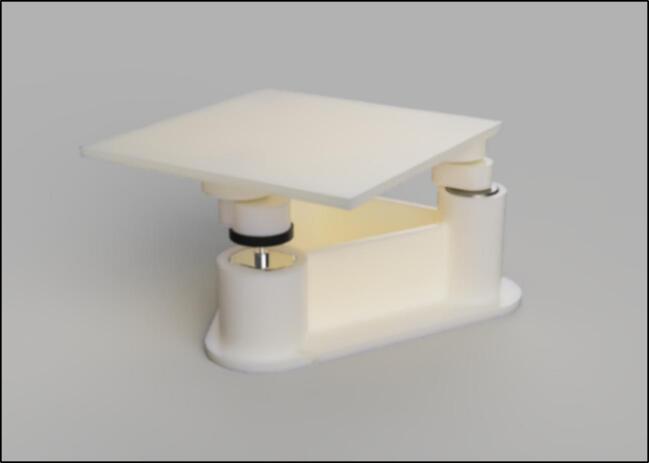
Open-source orbital shaker provided.

**Fig. 4:**
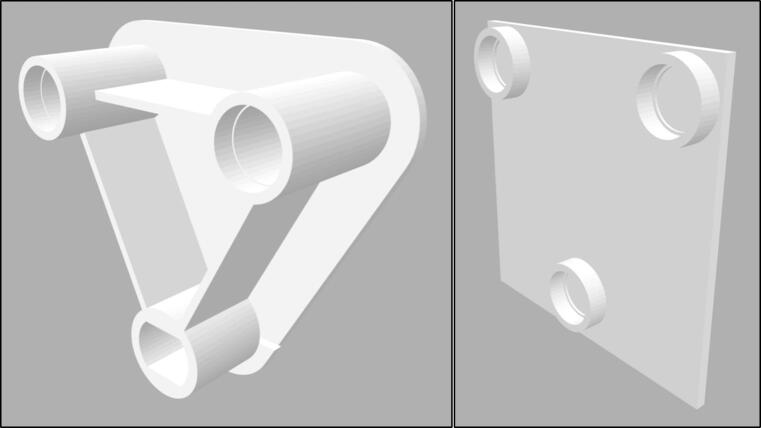
Base plate (left) and top plate (right).

The structure is made of four walls (30 × 30 × 5 cm) with an aluminium foil and insulant floor, with every surface that is exposed to the heater covered in foil. This simple structure enabled sufficient insulation up to 75 °C in a 23 °C temperature-controlled laboratory. Due to its inherent conductivity and the expulsion of air particles onto the foil, this can become statically charged, so it is crucial that all the aluminium foil is connected and grounded. The 3D-printed shaker is also covered in foil, but at low temperatures, the heat will cause minimal damage to the interlayer adhesions of the 3D-printed filament, so it is not required to be covered in foil. This may be important as damage has been recorded at low temperatures to 3D printed structures [21] (see Fig. 3, Fig. 4).

The motor is a 9 V motor part of the Arduino starter kit, which is powered by a 9 V DC supply. The design can be modified to accommodate different motors with different power requirements. The heating module is controlled by an Arduino microcontroller and consists of a commercial electric heater connected to a servo motor and a temperature sensor. The Arduino opens and closes the energy supply to the heater via a servo motor due to the input from the temperature sensor relative to the desired temperature. The servo is connected to the trip-shut-off switch on the heater. The trip-shut-off switch is a switch on the bottom of small heaters that turns the heater on and off when the heater is knocked over. This is important to note as the servo provided by Arduino does not have enough torque to turn the commercial heater switches on and off, whereas the trip-shut-off switch is easily turned on and off by a servo motor. This means the heater must be placed horizontally, and that the heating setting must be set before operating the experiment and maintained throughout. This enables the user to easily attach a servo without having to attach a servo directly to the heater temperature control. It is also noted that the heater used in the experiment, when set on the highest temperature, would trip its own heat damage control mechanism after around 2 min and shut itself off, so using the medium heat setting is recommended.

The temperature sensor used is a Ds18B20 stainless steel temperature sensor. Alternative sensors can be easily installed to provide more precise measurements, but it is noted that temperature is normally a small factor in the cause of error in these experiments. The Arduino starter kit does come with its own temperature sensor, but it was found to frequently malfunction during testing.

In summary, OpenHW3 hardware provides:

• Temperature control of scientific equipment in the range ‘Ambient to 75 °C’.
• Low-cost open-source Orbital Shaker ($169)
• Programmable temperature control
• Easy to assemble and operate.

Two open-source designs have been created, one in which an existing orbital shaker can be modified (MS2), and another where you can create your own orbital shaker (HW3). These designs are separated as laboratories may only require one of these components, and they may want to retrofit their current orbital shakers to be temperature controlled.

### 2.1 Design limitations

As a low-cost alternative to conventional systems, this system also has its own limitations. These limitations revolve around reliability for long term experimentation, and below ambient temperature control. For long term experimentation and critical data, using a commercial, high-end option is recommended. However, for temperature control ±1 °C, this can be achieved with OpenHW3 without any orbital shaking for the same specifications as a commercial option. The reliability issues arise when using any 3D printed moving parts long term – and this is seen within this project as the orbital shaking component.

### 2.2 Safety

Safety considerations are mentioned throughout the paper when relevant, however, some overall points are important to remember. Specifically – commercial alternatives will overall be safer than low-cost open-source alternatives like OpenHW3. The extent of this safety increase, however, relates mainly to the electric heater component. As OpenHW3 is blowing warm air towards insulant, aluminium foil, and 3D printed plastic component, the fire risk is low. However, although low, it is still present. It is for this reason OpenHW3 should be treated the same as running a low-cost electric heater anywhere – it could be run overnight, it could be run without supervision, but it would be much safer not to.

## 3 Design files summary

All CAD models were made in Fusion360, but files have been created for multiple model files, and are available in.stl for direct 3D printing without CAD software. The CAD files are built around the Arduino component sizes, and these standard sizes may not require much modification to the user. It is noted however that different 3D printers result in different tolerances, so users may have to make individual modifications to the design files. If using different temperature sensors, the specific library used to read temperatures from the sensor should be changed (see Table 3).

**Table 3:** Design file summary.

**Design file name**	**File type**	**Open source license**	**Location of the file**
For example: Design file 1	e.g., CAD files, figures, videos	All designs must be submitted under an open hardware license. Enter the corresponding open source license for the file.	Either enter the URL for the repository or the sentence: “Available with the article”.
Orbital shaker	CAD files (.f3d,.ipt,.skp,.stl)	CC0 1.0 Universal	https://osf.io/8n65f/
Code	.ino	CC0 1.0 Universal	https://osf.io/8n65f/
Build instructions	.pdf	CC0 1.0 Universal	https://osf.io/8n65f/
Operating instructions	.pdf	CC0 1.0 Universal	https://osf.io/8n65f/
Parts list	.pdf	CC0 1.0 Universal	https://osf.io/8n65f/

## 4 Bill of materials summary

**Table 4:** Bill of materials.

Designator	Component	Number	Cost per unit – $	Total cost – $	Source of materials	Material type
Ardu	Arduino uno starter kit	1	$110	$110	https://store.arduino.cc/products/arduino-starter-kit-multi-language	Micro controller with all electronics (including servo) included
Temp	Ds18B20 stainless steel temperature sensor	5	$10	$2	https://www.amazon.co.uk/Digital-Temperature-Ds18B20-Stainless-Accurate/dp/B07TKTFKMW/ref=sr_1_9?crid=37CMC2XNRF2OD&keywords=arduino+temperature+probe&qid=1675944693&sprefix=arduino+temperature+probe%2Caps%2C85&sr=8-9	Temperature sensor
Constr.	Tape or glue, foil	−	$7	$5	Local Hardware store (considerably lower cost than online)	Construction materials (Glue, tape, aluminum foil)
Heater	Commercial heater	1	$20	$20	https://www.amazon.com/Electric-Heaters-Portable-Thermostat-Heating/dp/B0895QVCYC/ref=sr_1_5?crid=GKLO7W6O7PL7&keywords=electric%2Bheaters&qid=1691059162&sprefix=electric%2Bheater%2Caps%2C179&sr=8-5&th=1	Heater
Inst	Insulant	1 m × 1 m	$5	$5	Local Hardware store (considerably lower cost than online)	Insulant for maintaining internal temperature
3D	3D printed components	5	$1	$5	https://osf.io/8n65f/	Orbital shaker structure
Bearing	Bearings	10	$5	$4	https://www.digikey.com/en/products/detail/mechatronics-bearing-group/608-2RS-W-CHEVRONSRI2/9608369	Enable rotation in the shaker
9VP	9 V mains power supply	1	$10	$10	https://www.amazon.co.uk/Charger-Adapter-Bluetooth-Speakers-Routers/dp/B08YJ94R31/ref=sr_1_9?crid=2OZ59JM024GOS&keywords=9v+DC+mains+supply&qid=1691059340&sprefix=9v+dc+mains+supply%2Caps%2C76&sr=8–9	Power the orbital shaker motor
TOTAL			$168	$157	(Incl. $12 Shipping)	$169

## 5 Build instructions

### 5.1 Orbital shaker construction

Two options (HW3 and MS2) are detailed for orbital shaking. Retrofitting a current orbital shaker (MS2) or utilizing the 3D printed design provided (HW3). These options are explained in the validation section as they relate to the results of the binding isotherms experiment. It is recommended to retrofit any existing shaker for reliability; however, the proceeding steps will detail how to fabricate your own HW3 orbital shaker.

The first step is purchasing and 3D printing the respective parts. These can all be found in the bill of materials and in the OSF repository (https://osf.io/8n65f/). All the files are open-source and can be easily edited to specific user requirements. It is recommended that the user edits the ‘top plate’ part to match the specific requirements of the beaker being used in the experiment.

### 5.2 3D printing parts

**Fig. 5:**
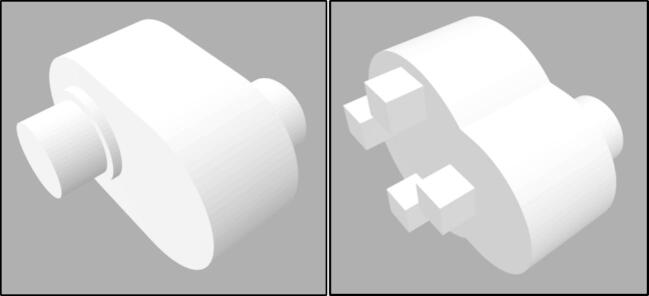
Bearing to bearing connector (left) and bearing to motor connector (right).

When 3D printing parts, it is recommended to select the highest setting (in terms of resolution, accuracy, and precision) available on the 3D printer. This may not be available or economical for all parts, but the ‘bearing to motor’ and ‘bearing-to-bearing’ connectors should be printed with the highest possible settings. The ‘bearing-to-bearing’ connector, displayed in Fig. 5, was made oversized as errors were reported with this part failing and bending over time [21].

### 5.3 Assembly of orbital shaker

**Fig. 6:**
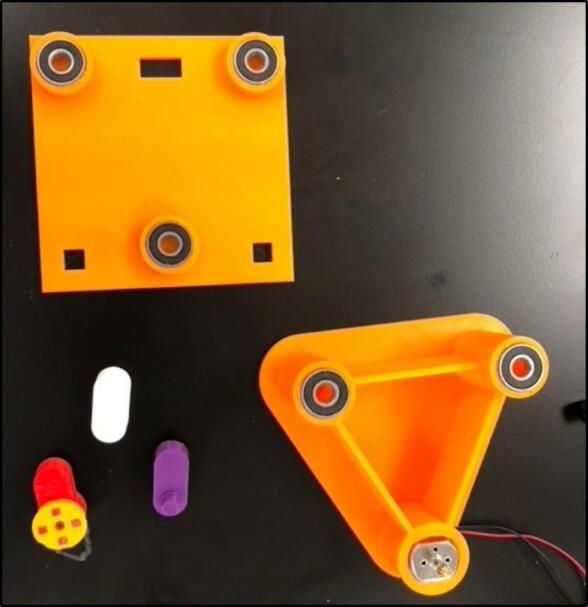
Base plate and top plate with bearings and motor fitted.

**Fig. 7:**
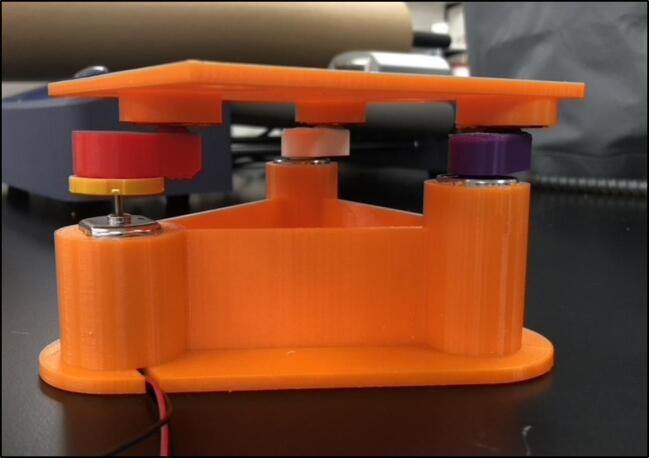
Full assembly of orbital shaker.

**Fig. 8:**
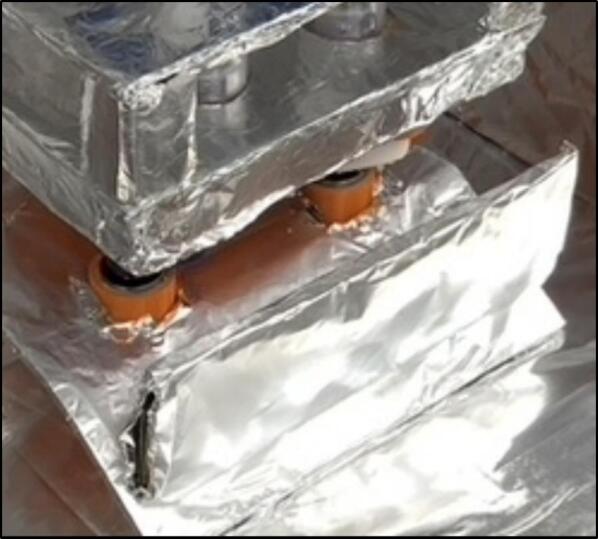
Makeshift heat shield demonstrated in operation.

When assembling the 3D printed orbital shaker, firstly connect the bearings and Arduino starter kit motor to the base plate. They are designed to all be press fits, however, due to variable tolerances of 3D printers, parts may need to be filed. Silicon hot glue can also be used if the bearings are loose in the base plate. Bearings are also attached to the top plate via a press fit (see Fig. 6, Fig. 7, Fig. 8).

Next, the ‘bearing-to-bearing’ and ‘bearing-to-motor’ connectors should be attached to the bearings, making sure they all face the same direction before you connect the top and bottom plates together. The connectors both attach via press fit, but for additional strength, glue can be used on the edge of the bearings.

### 5.4 Protective foil layer

**Fig. 9:**
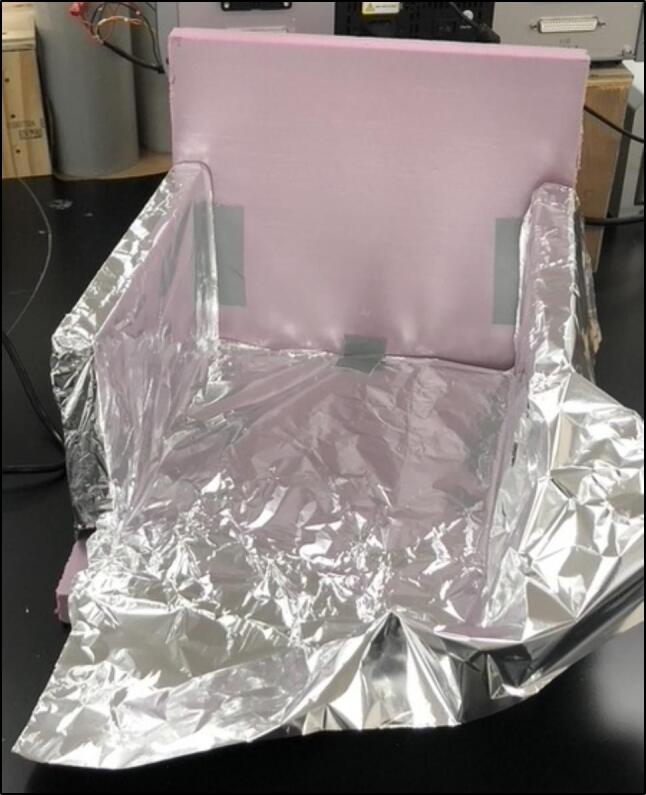
Insulating chamber under construction.

**Fig. 10:**
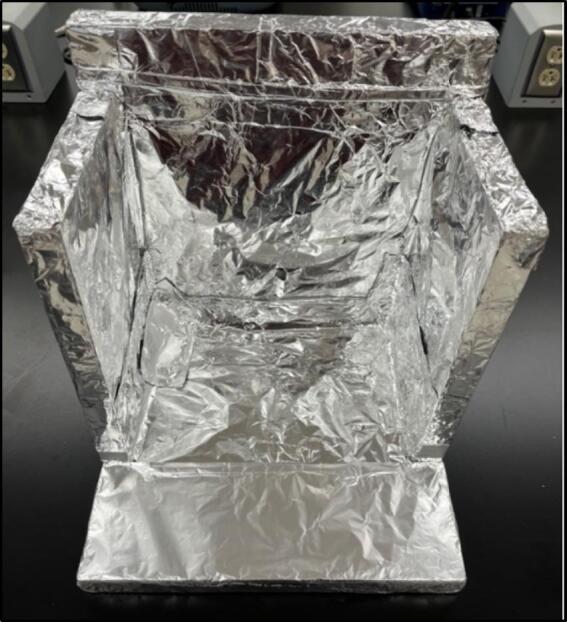
Complete insulating chamber.

As a precaution to prevent damage to the motor and the 3D printed shaker, exposed areas are covered in foil. A foil and scrap plastic heat shield were placed in front of the baseplate to shield it further from the heat. To this precaution, the motor was also placed furthest away from the heater, as to reduce the chance that overheating could occur. This foil placed however should not impede the movement of the shaker (see Fig. 9, Fig. 10).

### 5.5 Insulating chamber

**Fig. 11:**
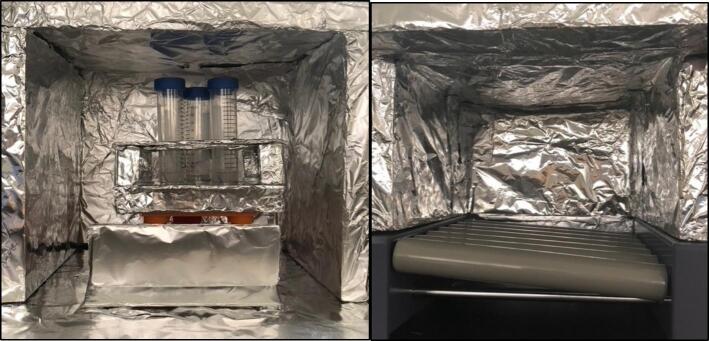
Complete insulating chamber over provided HW3 orbital shaker and commercial orbital shaker.

**Fig. 12:**
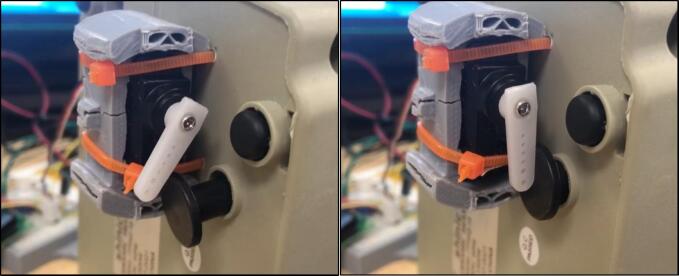
Range of movement required from the servo pressing the button.

The insulating chamber maintains the temperature produced from the heater to help control the temperature. The chamber is constructed by assembling a frame of four insulated walls fitting the specification of the orbital shaker or device it is covering. If the provided orbital shaker is used, then the sides must extend the width of the heater and the height of the orbital shaker plus the container used in the proposed experiment. This may typically be around 25–30 cm. The insulant walls in this example were attached together with hot glue and tape, but other methods can be used as needed. If you are using the orbital shaker provided, then a 30 × 30 × 30 cm box meets the requirements. It is important to note that a mask should be worn when cutting insulation as it may contain harmful pollutants (see Fig. 11, Fig. 12).

Foil is used to cover all the internal surfaces of the walls, including all insulant and any surfaces that face the electric heater in any way. The foil was attached with tape, and specific effort was made to make sure no tape was in contact with the hot air from the heater. When the insulating chamber is constructed, a small hole is cut into the roof piece of the chamber in the middle. This was done with a pencil (as the insulant used was very brittle) and is for the temperature sensor to slot into. A pencil is a good diameter for the sensor, but if a drill is required, then a 8 mm diameter drill is recommended. The temperate sensor can be placed inside the hole and placed such that no plastic on the sensor is within the chamber.

### 5.6 Retrofitting the heater

Retrofitting the heater requires attaching the servo in a position where it will toggle the trip shut-off on the heater. This feature is available on the heater indicated and is specifically for shut off when the heater falls over. This was chosen over directly tampering with the internal power electronics or the main on/off switch. The main on/off switch requires a large amount of force to turn, and the Arduino provided servo does not have the power to do so. This requires the heater to be raised off the ground, as orbital shakers normally operate between 10 cm and 20 cm above ground level. To hold the switch in place, an improvised servo holder was made from spare 3D printed parts.

Regarding safety, this stage and process possesses the majority of the risks associated with OpenHW3. As a trip shut-off is used instead of the main power switch, there are pros and cons. Cons, obviously are that you are removing a main safety feature of the heater. Although the heater falling over is a minimal risk, the feature is still present for a reason. This factor is to be considered before operating this product. Pros however, mean that neither the power switch, overheating switch, or mains supply is tampered with. Tampering and remotely controlling these are significantly more hazardous, and should be avoided.

With the heater used in this experiment, there are two heat and fan settings. This is detailed in the validation, where the highest heat setting triggers the heaters built-in maximum temperature safety switch after around 2–3 min of use, so the middle heat setting was used and the fan setting for cooling the heater.

### 5.7 Circuit construction

**Fig. 13:**
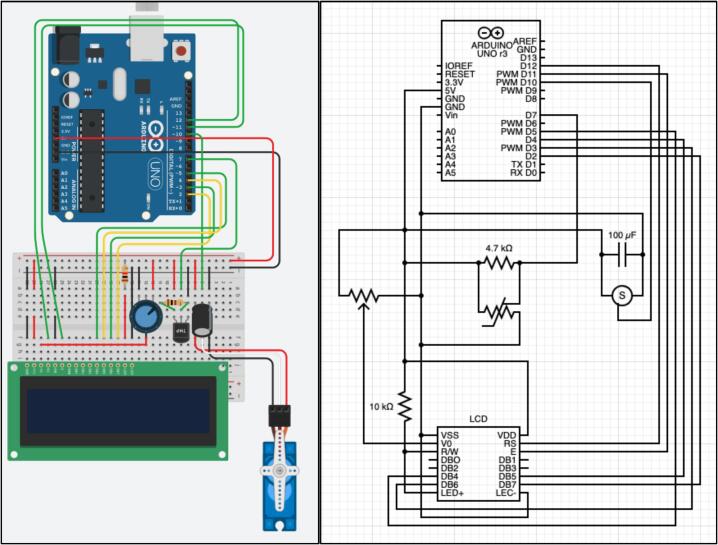
Circuit design for OpenHW3.

The circuit was constructed with the Arduino starter kit as displayed in Fig. 13. In the diagram, certain colour wires are used, however all the wires in the starter kit are identical in conductive properties, only the wire length varies with colour. It is noted the green and yellow wires are longest and are the only ones that reach the ‘digital PWM’ output nodes. For the full diagram schematic, the TinkerCAD file is available through https://www.tinkercad.com/things/5EtaIR1Vgui.

The circuit reads and controls the temperature, and displays this information to the user. It does this by utilizing an LCD display, servo motor and temperature sensor. These are all connected directly to the Arduino in the various standard configurations displayed in the Arduino starter manual. The LCD will display the current temperature and the goal temperature for the chamber. This is connected to specific ports in the Arduino, with a potentiometer used to control the brightness of the display screen. Additionally, the Servo motor is attached to the heater component. A 3-pin connector was used (available in the starter kit) to connect the servo to the breadboard by slotting into the servo wiring connector. The servo is then attached to a capacitor, which is indicated in the Arduino starter manual as to which is the correct capacitor for the servo.

The temperature sensor used is not provided with the Arduino starter kit, but rather is displayed in the bill of materials in Table 4. This is because the sensor in the starter kit does not have the same accuracy or protection to heat. The resistor used to connect the temperature sensor comes with the temperature sensors indicated. Different temperature sensors can be used, and the resistors provided with them should be used. It is also important to note it is much easier to wire the circuit using the breadboard provided in the starter kit with small wire connectors. This helps to keep the circuit visually easy to work with and makes it easier to slot wires into place. Additionally, bending the end of the wires is not advised, as their connections become weaker to the breadboard and causes connection errors.

### 5.8 Software

The software operates by displaying to the user via LCD display the current temperature and goal temperature. This corresponds simply to an if/else statement that when the temperature of the chamber is too high, the heater turns off, and if the temperature is too low it switches on. This method was chosen over the use of PID control because it was found that accuracy can be achieved with this simple set up and software to ±1 °C up to 75 °C, so no further control was required. PID control can be implemented in this system and is recommended for more precise control, but it will be chamber and heater specific. Additionally, two libraries are included called ‘DallasTemperature.h’ and ‘OneWire.h’. These are used to read the temperate sensor in the parts list.

### 5.9 Entire construction

**Fig. 14:**
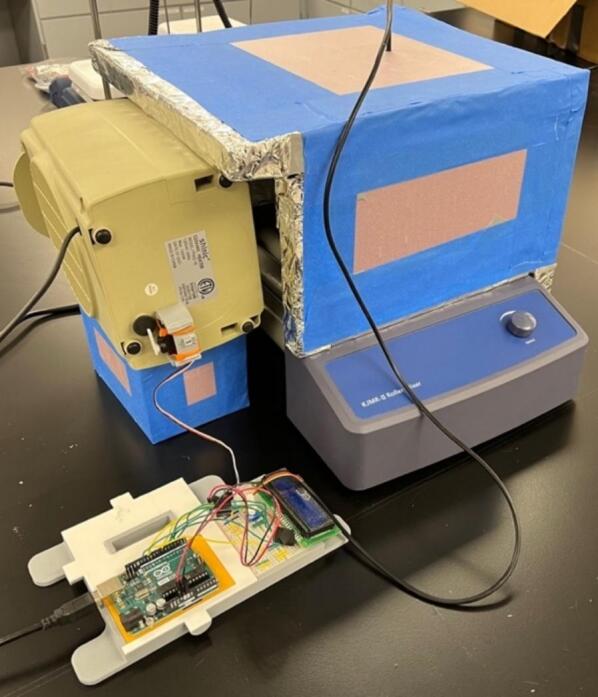
Full assembly with an existing orbital shaker, noting the heater raised off the ground and the Arduino away from the heat.

**Fig. 15:**
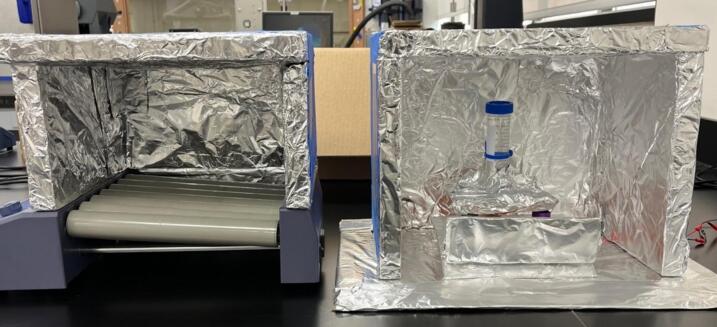
Both chamber options displayed.

**Fig. 16:**
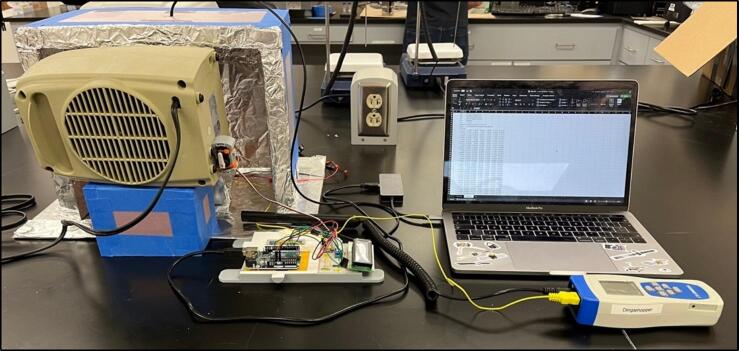
Experimental set up for temperature validation and variable temperature programming.

The final assembly involves connecting all the components into the desired configuration (see Fig. 14, Fig. 15, Fig. 16). Before starting, make sure the Arduino is far away from any heat being blown into the system, as to avoid any damage. Additionally, make sure the motor (if used in the orbital shaker) is protected from heat to prevent overheating. Also, the heater should be close enough to the insulating chamber, and is at an appropriate height, so it can efficiently heat the specimen without overheating the orbital shaker. For most orbital shakers, and the one provided, that will be 10 cm off the ground. Finally, make sure you are in a safe environment before you proceed to the operating instructions, as there is a present fire risk when using low- cost electric heaters.

## 6 Operation instructions

Firstly, to safely operate OpenHW3, it is important to ensure that the heater and servo are not plugged in. Plug the Arduino into the mains, connect a laptop and run the code to make sure the LCD is displaying properly, the temperature sensor is reading values, and the servo moves the button on and off. The main reason issues may develop in this initial step are incorrect wire connection and incorrect adjustment of the potentiometer connected to the LCD.

Secondly, connect the servo and run the code again at a higher temperature to see if the servo will move in the correct direction when it is turned on or off. If the servo is in the incorrect position, change the angles at which the servo operates so that when the heater is required to be turned on, the on-button for the heater is engaged properly, and when it is turned off, the servo releases the heater button.

With these checks completed, make sure the beakers used in the experiment fit on top of the orbital shaker with appropriate clearance. Ensure that its motion can be maintained by the shaker and will not collapse or cause resonance with the shaker.

The motor is operated in the orbital shaker by simply connecting it to the 9 V AC-DC mains power supply, and not to the Arduino. It is possible to connect it to the Arduino and facilitate PWM speed control, however, there is not enough space on the breadboard to facilitate this simply. It should operate continuously without any undue disturbance. If there are issues with its rotation, a common cause can be the alignment of the ‘bearing-to-bearing’ connectors to each other and the ‘bearing-to-motor’ connectors. These three components should be perfectly parallel to each other.

Before turning the heater on, it is suggested to run the heater with just air and no heat to blow out any dust in the heater. After clearing out the heater, calibrate the temperature sensor by running the code (with the heater turned off) to align the room temperature with the temperature read by the Arduino. This was completed by using a laboratory sensor and manually adjusting the code to align the values.

Next, the heater should be set to a number (30–40 °C) and run for at least 10 min. This value should be monitored by a laboratory temperature sensor, to further calibrate the scaling of temperature. With this, it is noted that there is a delay between the precise temperature measured in the heater and the temperature record by the sensor. This is due to the metal protective layer around the sensor requiring time to conduct heat through to the sensors beneath. This delay is modelled to be around 30–40 s, with the exact air temperature spiking before levelling out to the required temperature. It is this effect that means you should allow time for the temperature to level to the goal temperature before you undertake any experiment with the equipment. With the temperature calibrated, it is still worth taking random temperature measurements throughout the experiment.

If you wish to stop the experiment at any time, the easiest way to halt all equipment is to turn it off at the mains, or alternatively if you just want to stop the heat and not the orbital shaking motion, turn the heater from hot air to either off or just air. Just air (normally indicated by a fan icon on the heater), is a useful setting as it disperses the air inside the heater and cools the insulating chamber considerably quicker than just turning the heater off. However, the latent heat in the heater means that it will continue to pump warm air out for around 5–10 s before being significantly cooler.

## 7 Validation and characterization

### 7.1 Temperature control

**Fig. 17:**
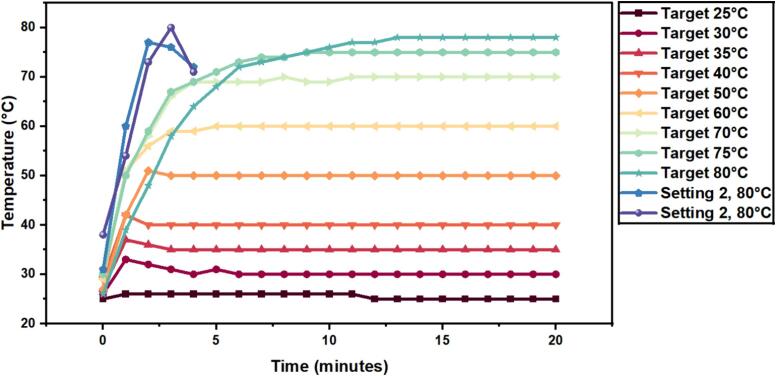
Temperature control of OpenHW3.

Displayed in Fig. 17, temperature equilibrium in the OpenHW3 can be reached after 12 min for the range of temperatures shown (25–75 °C). The heating times are variable and increase proportional to the increase in target temperature, however just above room temperature (25 °C) was difficult to stabilize due to the high heat produced by the heater which were compensated with long cooling periods and sharp increases in heat. This could be counteracted using PID control; however, this is very close to laboratory conditions so heating may not be required. At target temp 80 °C, Fig. 17 shows 2 curves that both stop at 4 min. This is because a higher heater setting was used compared to the other temperature, and as a result the heaters temperature safety limit was reached and the heater automatically shuts off, and for this heater setting that happened at around 2–3 min. This is specific to the heater used but may be common if other low-cost commercial heaters are used. Additionally, target temp 80 °C was unable to be reached at the heat setting used for 25–75 °C.

### 7.2 Binding isotherm experimental results

To test the effectiveness of OpenHW3, a binding isotherm experiment was carried out following the methodology by Jain et al. [23]. A novel Mg-Al-P cementitious material (based upon work by Reed et al. [23]) being evaluated for nuclear waste containment was used to determine its binding isotherm with strontium diluted with 1 M nitric acid. While the precise results are not relevant to the validation, the variation of isotherms changes as related to a control are used to provide basis for the validation of accuracy in the OpenHW3 apparatus. The variation in strontium content were determined utilizing Inductively Coupled Plasma Emission Spectrometry (ICP-AES). The mixing methods explored were manual mixing, where at room temperature a sealed container is hand mixed every 5 min for 10 s (Manual Mixing), OpenHW3 orbital shaker (OpenHW3), a commercial horizontal roller shaker repeated twice (Horizontal and Heated Horizontal), and a commercial water bath (Waterbath). The control is represented by Manual Mixing (which is an unfavourable no equipment option) and the Waterbath (which is a favourable, high-cost experimental option). 0.1 mL of solution will be extracted from the containers at periodic intervals, where upon collection it will be mixed with diluted nitric acid before ICP-AES. Sample 129 represents a novel Mg-Al-P cementitious material.

**Fig. 18:**
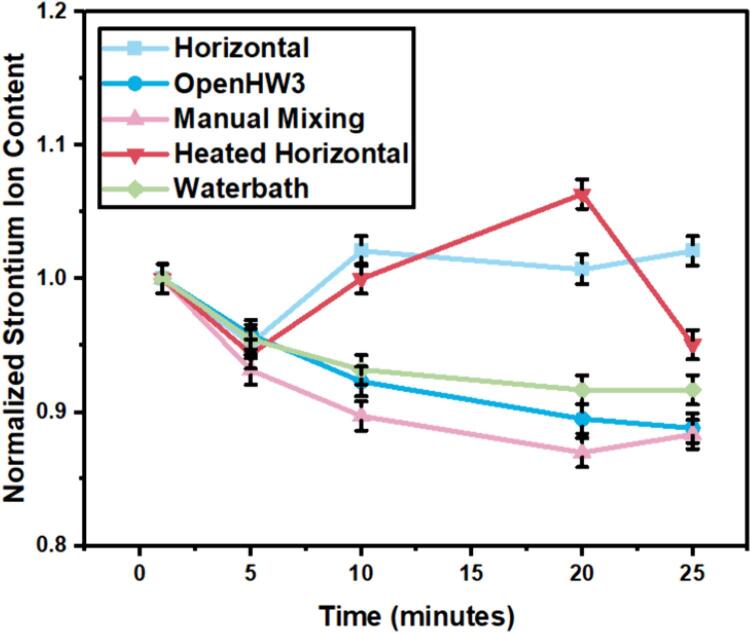
Change in strontium content for binding isotherm experiment.

As displayed in Fig. 18, the variation in strontium content changes significantly in the Horizontal and Heated Horizontal set ups. These both represent the same type of horizontal shaking motion. OpenHW3 and the Waterbath both represent vertical orbital shaking motion, with the same 1.9 mm orbital radius. They both are observed to have similar characteristics. It is also noted that the ratio change attained with Manual Mixing does not vary widely when compared to the Waterbath and OpenHW3 options to perform this binding isotherm experiment. This is due to the Manual Mixing shaking motion which occurred every 5 min after a sample was taken. However, for long duration experiments, this method may not be practical and requires a larger time investment.

The variability in Horizontal and Heated Horizontal set ups compared to vertical orbital shaking options is attributed to the plane at which the mix-shaking occurs. This parameter is observed to be critical in the binding isotherms experiment, as shown by the higher ratio change in strontium for both Horizontal and Heated Horizontal set ups in Fig. 18. Visual inspection of the samples show the formation and aggregation of precipitates in the solution. This precipitate aggregation has been attributed to improper mixing of the solid material sample throughout the beaker and which normally remains in the bottom section of the beaker. Samples are typically extracted from the top section of the beaker, so this will not affect sampling if the beaker remains vertical. However, when horizontal mixing is utilized, this floating precipitate aggregate may be sampled from the top section. This sampling of floating aggregate may dramatically impact the ion content detected by ICP.

With this, OpenHW3 shows similar results to the Waterbath and manual mixing. Overall, combining both the temperature control and binding isotherm experimental results, OpenHW3 could perform the same functionality as conventional temperature-controlled water bath shakers, for a significant reduction in cost.

## 8 Conclusions and discussion

OpenHW3 presents an open-source orbital shaker that can control temperature from ambient to 75 °C. Additionally, OpenHW3 provides an alternative way to temperature control scientific equipment without the significant rise in costs associated with temperature-controlled scientific equipment. Additionally, using open-source equipment and software, significant possibilities for improvements and connection to the Internet of Things (IOT) for remote control and use are available. Improvements can be made to this design, however, greater improvements result in a higher cost, a more complex assembly, and ultimately more time being spend to make the equipment function, which approaches a diminishing return on investment the closer that combined cost comes to a commercial equivalent. With these considerations, OpenHW3 does not replace commercial heated orbital shakers, but provides a significantly lower cost option to ensure a high replicability of binding isotherms experiments.

## CRediT authorship contribution statement

**Matthew Hollingham:** Writing – original draft, Methodology, Investigation, Conceptualization. **Yi Xiang:** Writing – review & editing, Methodology, Data curation. **Titus Reed:** Resources, Project administration, Conceptualization. **Juan Pablo Gevaudan:** Writing – review & editing, Supervision.

## Declaration of competing interest

The authors declare that they have no known competing financial interests or personal relationships that could have appeared to influence the work reported in this paper.

## References

[1] Jain A., Gencturk B., Pirbazari M., Dawood M., Belarbi A., Sohail M.G., Kahraman R. (2021). Influence of pH on chloride binding isotherms for cement paste and its components. Cem. Concr. Res..

[2] Yuan Q., Shi C., De Schutter G., Audenaert K., Deng D. (2009). Chloride binding of cement-based materials subjected to external chloride environment – A review. Constr. Build. Mater..

[3] H. Ye, X. Jin, W. Chen, C. Fu, N. Jin, Prediction of chloride binding isotherms for blended cements, Comput. Concr, 17(5) (2016) 655-672, 10.12989/cac.2016.17.5.655.

[4] Martín-Pérez B., Zibara H., Hooton R.D., Thomas M.D.A. (2000). A study of the effect of chloride binding on service life predictions. Cem. Concr. Res..

[5] M. Yan, O. Ramström, Molecularly Imprinted Materials, Science and technology, Chapter 16, Binding Isotherms, 2004, ISBN 78-1-4200-3030-3, https://doi.org/10.1201/9781420030303.

[6] Jonathan B Chaires, Analysis and interpretation of ligand-DNA binding isotherms, Methods in Enzymology, Academic Press, Volume 340, 2001, Pages 3-22, ISSN 0076-6879, ISBN 9780121822415, https://doi.org/10.1016/S0076-6879(01)40415-0. 10.1016/s0076-6879(01)40415-0 11494856

[7] Misra V.K., Draper D.E. (1999). The interpretation of Mg2+ binding isotherms for nucleic acids using Poisson-Boltzmann theory1 1Edited by B. Honig. J. Mol. Biol..

[8] Crothers D.M. (1968). Calculation of binding isotherms for heterogeneous polymers. Biopolymers.

[9] Lapitsky Y. (2007). Calorimetric determination of surfactant/polyelectrolyte binding isotherms. J. Phys. Chem. B.

[10] Puziy A.M., Poddubnaya O.I., Martínez-Alonso A., Suárez-García F., Tascón J.M.D. (2002). Synthetic carbons activated with phosphoric acid: I. Surface chemistry and ion binding properties. Carbon.

[11] Tritthart J. (1989). Chloride binding in cement II. The influence of the hydroxide concentration in the pore solution of hardened cement paste on chloride binding. Cem. Concr. Res..

[12] Luping T., Nilsson L.-O. (1993). Chloride binding capacity and binding isotherms of OPC pastes and mortars. Cem. Concr. Res..

[13] Arya C., Buenfeld N.R., Newman J.B. (1990). Factors influencing chloride-binding in concrete. Cem. Concr. Res..

[14] Anik Delagrave, Jacques Marchand, Jean-Pierre Ollivier, Simone Julien, Kati Hazrati, Chloride binding capacity of various hydrated cement paste systems, Advanced Cement Based Materials, Volume 6, Issue 1, 1997, Pages 28-35, ISSN 1065-7355, https://doi.org/10.1016/S1065-7355(97)90003-1.

[15] Iqbal M., Schiewer S., Cameron R. (2009). Mechanistic elucidation and evaluation of biosorption of metal ions by grapefruit peel using FTIR spectroscopy, kinetics and isotherms modeling, cations displacement and EDX analysis. J. Chem. Technol. Biotechnol..

[16] Brian P. (2000). Spalding, volatility and extractability of strontium-85, cesium-134, cobalt-57, and uranium after heating hardened portland cement paste. Environ. Sci. Tech..

[17] Dada, A, Olalekan, A, Olatunya, A, DADA, O, Langmuir, Freundlich, Temkin and Dubinin–Radushkevich Isotherms Studies of Equilibrium Sorption of Zn2+ Unto Phosphoric Acid Modified Rice Husk, Journal of Applied Chemistry (IOSR-JAC) ISSN: 2278-5736. Volume 3, Issue 1(Nov. – Dec. 2012), PP 38-45, www.iosrjournals.org.

[18] K. Behere, S. Yoon, n-Layer BET adsorption isotherm modeling for multimeric Protein A ligand and its lifetime determination, J. Chromatogr. B, 1162, 2021, 122434, ISSN 1570-0232, https://doi.org/10.1016/j.jchromb.2020.122434. 10.1016/j.jchromb.2020.122434 33302227

[19] DIYbio Orbital Shaker V 1.0 by ProgressTH, Available at https://www.thingiverse.com/thing:2633507, 2018.

[20] Open Source orbital shaker, available at https://www.thingiverse.com/thing:5045, 2010.

[21] Baillargeon P., Fernandez-Vega V., Ortiz L., Shumate J., Marques N., Deng L., Spicer T.P. (2022). Louis Scampavia, Rapid deployment of inexpensive open-source orbital shakers in support of high-throughput screening. SLAS Technology.

[22] C. Sánchez, P. Dessì, M. Duffy, Piet N.L. Lens, OpenTCC: An open source low-cost temperature-control chamber, HardwareX, Volume 7, 2020, e00099, ISSN 2468-0672, https://doi.org/10.1016/j.ohx.2020.e00099. 10.1016/j.ohx.2020.e00099 PMC9041232 35495215

[23] Reed T., Mauro J.C., Gevaudan J.P. (2024). Influence of phosphates on phase formation in alkali-activated MgO-Al2O3-SiO2-P2O5 cements. Int. J. Ceram Eng. Sci..

